# Epigenetic modifying enzyme expression in asthmatic airway epithelial cells and fibroblasts

**DOI:** 10.1186/s12890-017-0371-0

**Published:** 2017-01-31

**Authors:** Dorota Stefanowicz, Jari Ullah, Kevin Lee, Furquan Shaheen, Ekiomoado Olumese, Nick Fishbane, Hyun-Kyoung Koo, Teal S. Hallstrand, Darryl A. Knight, Tillie-Louise Hackett

**Affiliations:** 1 UBC Centre for Heart Lung Innovation, St. Paul’s Hospital, 1081 Burrard Street, Vancouver, BC V6Z 1Y6 Canada; 2 Department of Biological Sciences, Southern Methodist University, Dallas, TX USA; 3 Department of Medicine, Division of Pulmonary and Critical Care, University of Washington, Seattle, USA; 4 School of Biomedical Sciences and Pharmacy, Faculty of Health and Medicine, University of Newcastle, Callaghan, NSW Australia; 5 Department of Anesthesiology, Pharmacology and Therapeutics, University of British Columbia, Vancouver, BC Canada

**Keywords:** Asthma, Histone modification, Epigenetics, Airway epithelium, Airway epithelial cells, Airway Fibroblasts, Epigenome, Histone code, Post-translational modification, DNA methylation

## Abstract

**Background:**

Recognition of the airway epithelium as a central mediator in the pathogenesis of asthma has necessitated greater understanding of the aberrant cellular mechanisms of the epithelium in asthma. The architecture of chromatin is integral to the regulation of gene expression and is determined by modifications to the surrounding histones and DNA. The acetylation, methylation, phosphorylation, and ubiquitination of histone tail residues has the potential to greatly alter the accessibility of DNA to the cells transcriptional machinery. DNA methylation can also interrupt binding of transcription factors and recruit chromatin remodelers resulting in general gene silencing. Although previous studies have found numerous irregularities in the expression of genes involved in asthma, the contribution of epigenetic regulation of these genes is less well known. We propose that the gene expression of epigenetic modifying enzymes is cell-specific and influenced by asthma status in tissues derived from the airways.

**Methods:**

Airway epithelial cells (AECs) isolated by pronase digestion or endobronchial brushings and airway fibroblasts obtained by outgrowth technique from healthy and asthmatic donors were maintained in monolayer culture. RNA was analyzed for the expression of 82 epigenetic enzymes across 5 families of epigenetic modifying enzymes. Western blot and immunohistochemistry were also used to examine expression of 3 genes.

**Results:**

Between AECs and airway fibroblasts, we identified cell-specific gene expression in each of the families of epigenetic modifying enzymes; specifically 24 of the 82 genes analyzed showed differential expression. We found that 6 histone modifiers in AECs and one in fibroblasts were differentially expressed in cells from asthmatic compared to healthy donors however, not all passed correction. In addition, we identified a corresponding increase in Aurora Kinase A (AURKA) protein expression in epithelial cells from asthmatics compared to those from non-asthmatics.

**Conclusions:**

In summary, we have identified cell-specific variation in gene expression in each of the families of epigenetic modifying enzymes in airway epithelial cells and airway fibroblasts. These data provide insight into the cell-specific variation in epigenetic regulation which may be relevant to cell fate and function, and disease susceptibility.

**Electronic supplementary material:**

The online version of this article (doi:10.1186/s12890-017-0371-0) contains supplementary material, which is available to authorized users.

## Background

Asthma is a chronic inflammatory condition of the airways that affects around 300 million people worldwide [1]. The airway epithelium, derived from the endoderm, is the first structural barrier to the inhaled environment in the airway mucosa. In asthma, the airway epithelium has an altered phenotype displaying altered cell cycle kinetics and increased numbers of basal cells [2, 3]. In addition, the lamina propria of asthmatic donors contains resident fibroblasts, derived developmentally from the mesoderm, that have been shown to exhibit an invasive and synthetic phenotype [4–8]. How these alterations in cellular phenotype occur in the disease is unknown but it is clear from the many genomic studies that asthma involves both genetic and environmental components.

The epigenetic landscape is essential in determining cell fate through histone modification and DNA methylation patterns that regulate the expression of genes integral to cellular development and differentiation [9–11]. Covalent modifications of the histone N-terminal tails can regulate gene expression and include acetylation, methylation, phosphorylation, and ubiquitination [12, 13]. Histone acetylation and phosphorylation are associated with a more open chromatin structure and gene expression, whereas histone methylation and ubiquitination can work both in a gene repressive and expressive manner depending on the target residue [13–17]. The enzymes responsible for the addition/removal of these modifications include: histone acetyltransferases (HATs)/deacetylases (HDACs), protein kinases/phosphatases, histone methyltransferases (HMTs)/demethylases (HDMs), and ubiquitin ligases/deubiquitinating enzymes (DUBs) [13, 17]. DNA methylation is facilitated by DNA methyltransferases (DNMTs) that add a methyl group to cytosine bases, forming 5-methylcytosine (5-mC) [12]. Addition of this mark at a gene promoter is generally associated with transcriptional repression and gene silencing [12, 18]. Furthermore, the epigenome is adaptable; it has the capability to respond to and be modified by environmental factors [10]. The outcome of this interaction depends on the environmental stressor and can be a normal physiological response or deregulation of the epigenome producing an abnormal phenotype [10, 19].

Abnormal epigenetic control of gene expression has been identified in both fibroblasts and epithelial cells in numerous pathologies [20–25]. However, very little is known about the expression and regulation of epigenetic modifying enzymes in asthma. Indeed, dysregulation of epigenetic mechanisms in asthma has been identified in a variety of cells but most studies have been performed in tissues from outside of the lung [26]. While dysregulation of enzymes involved in histone acetylation was identified in the airways of asthmatics, there is still disagreement on the exact enzymes responsible [27–30]. We have additionally identified unique DNA methylation patterns in airway epithelial cells (AECs) from asthmatic donors [31] yet research on the variability of the enzymes responsible for these changes is lacking.

To further elucidate the mechanisms driving the epigenetic alterations observed in the asthmatic airways, a better understanding of the gene expression profiles of epigenetic modifying enzymes in airway tissues is required. We hypothesize that the gene expression of epigenetic modifying enzymes is cell-specific and influenced by asthma status in tissues derived from the airways. Specifically, the aim of this study was to identify if the expression profiles of epigenetic modifying enzymes is cell- and disease-specific by profiling 82 genes across 5 families of epigenetic enzymes in AEC and fibroblasts from healthy and asthmatic donors. We identified 24 cell-specific and 7 disease-specific differentially expressed genes (6 in AECs and one in fibrolasts). Although not all of the disease-specific genes passed correction, we were able to identify a corresponding change in AURKA protein expression in asthmatic compared to healthy individuals.

## Methods

### Sample collection

AECs and airway fibroblasts obtained from de-identified human lungs from asthmatic and healthy donors not suitable for transplantation and donated for medical research were obtained though the International Institute for the Advancement of Medicine (Edison, NJ). A lung was identified as healthy if the donor had no history of asthma or other pulmonary disease or damage. Conducting airways down to the 5^th^ generation were used for AECs isolation by pronase digestion and airway fibroblasts were obtained by outgrowth technique as previously described [32, 33]. Endobronchial airway brushings from patients were also used to obtain AECs as previously described [34, 35]. AECs were grown in Bronchial Epithelial Growth Medium (BEGM, Lonza, Walkersville, MD) containing 100U/mL penicillin and 100ug/mL streptomycin, whereas fibroblasts were grown in Dulbecco’s Modified Eagle’s medium (DMEM) (Invitrogen, Burlington, ON, Canada) supplemented with 10% FBS, 2 mM L-glutamine, and 1% antibiotic/antimycotic solution. Cultures were maintained at 37 °C in a humidified 95% air/5% CO_2_ atmosphere to passage 2. Donor demographics are provided in Table 1.

**Table 1:** Donor demographics including disease status, age, cell type, and sex

Cell type	Disease status	Number	Cell source (W/B)	Sex (M/F)	Average Age° (range)
AEC	Asthmatic	11	7/4	5/6	18.8 (8–29)
	Healthy	13	8/5	6/7	22.6 (11–42)
Fb	Asthmatic	6	6/0	6/0	20.8 (10–36)
	Healthy	6	6/0	6/0	18.2 (5–43)

Airway epithelial cells (AECs) and airway fibroblasts (Fb) were collected from healthy and asthmatic donors. Cell source is identified by whole lung (W) or brushing (B). There were no differences for age between all groups by one-way ANOVA; *p* = 0.69

### Gene expression

AECs and airway-derived fibroblasts were grown in 6-well plates to 80% confluence, at which point RNA was collected using RNeasy Mini Kits (Qiagen). 500 ng of RNA was used to synthesize cDNA using the RT^2^ First Strand Kit (Qiagen). cDNA was then combined with 2× RT2 SYBR Green Mastermix (Qiagen) and RNase-free water and distributed onto a manufacturer optimized 384-well Human Epigenetic Chromatin Modification Enzymes Focused Array (PAHS-085E-4, Qiagen) pre-loaded with primers targeting 84 genes encoding epigenetic enzymes and 5 housekeeping genes as per manufacturer’s protocol. A complete list of the genes that were analyzed is available in Table S1 (Additional file 1). Additionally, to identify gene expression of CREBBP and EP300, cDNA was combined with 2× RT2 SYBR Green Mastermix, RNase-free water, and primers targeting CREBBP (PPH00324F-200, Qiagen), EP300 (PPH00319A-200, Qiagen), hypoxanthine phosphoribosyltransferase 1 (HPRT1, PPH01018C, Qiagen), ribosomal protein L13a (RPL13A, PPH01020B-200, Qiagen), and glyceraldehyde-3-phosphate dehydrogenase (GAPDH, PPH00150F, Qiagen) and loaded onto 384-well reaction plates. Data cleaning and housekeeping gene selection is described in Supplementary Methods (Additional file 2). Target gene expression was calculated using the delta Ct method: 2^(C_t_Housekeeping Gene – C_t_Target Gene)*10000.

### Sodium dodecyl sulfate polyacrylamide gel electrophoresis (SDS-PAGE) and immunoblot

Protein was collected from AECs and airway fibroblasts in culture and electrophoresed on a 12.5% SDS-polyacrylamide gel. Membranes were first incubated overnight with primary antibody (Table 2), then with goat anti-mouse IR-800 (1:2500, Vector Laboratories) or goat anti-rabbit Alexa 680 (1:2500, Invitrogen) secondary antibody, and finally imaged on the LI-COR Odyssey system. Odyssey software 1.1 was used to perform densitometry (LI-COR Biotechnology, Lincoln, NE, USA). Data for AURKA and SMYD3 were normalized to β-tubulin and hsp-90 respectively. A two-tailed unpaired *t*-test was performed, a *p*-value of less than 0.05 was considered significant.

**Table 2:** Antibodies used in experiments

Epitope	Host	Company	Catalogue number	Primary antibody dilution
SMYD3	Rabbit	Abcam	ab155018	1/1000
AURKA	Mouse	Cell Signaling	12100	1/500
Hsp90	Mouse	BD Biosciences	610418	1/1000
β-tubulin	Mouse	Millipore	05–661	1/2000
CREBBP	Rabbit	Santa Cruz Biotechnology	sc-369	1/50

### Immunohistochemical staining

Airway sections were formalin fixed and paraffin embedded prior to immunohistochemical staining. Sections were deparaffinized, rehydrated, processed for antigen retrieval and incubated overnight at 4 °C with CREBBP antibody (Table 2). Sections were subsequently incubated with a biotinylated goat anti-rabbit secondary antibody (1:100, Vector Laboratories, Burlingame, CA, USA) prior to visualization with Streptavidin-HRP (Dako) and 3,3-diaminobenzidine (Dako). Slides were counterstained with Harris Hematoxylin solution (Sigma, St. Louis, MO, USA) and dehydrated before coverslipping with Cytoseal 60 medium (Richard-Allan Scientific, Kalamazoo, MI, USA).

Using the Nikon Eclipse 700 (Nikon Instruments, Melville, NY, USA) with a 60× objective and SPOT Advanced software (Diagnostic Instruments, Sterling Heights, MI, USA), five images were obtained from each section. These images were analyzed for positively and negatively stained nuclei using ImagePro Plus software (Media Cybernetics, Rockville, MD, USA).

### Principal component analysis (PCA)

Principal components analysis was performed to assess sources of variation in our gene expression dataset. Principal components were obtained as a new set of orthogonal variables by extracting eigenvectors from singular value decomposition of the expression matrix, and ranked by the size of their respective eigenvalue, representing the component of overall variation.

### Co-expression analysis

Co-expression analysis was performed by calculating Spearman correlations between genes using pairwise-complete observations. This correlation matrix was then used to generate heatmaps showing co-expression between genes. Using the correlation matrix, we calculated the effective number of independent variables (ENIV) in each data set using spectral decomposition [36]. For analyses using both AECs and airway fibroblasts, only AECs, and only airway fibroblasts, the adjustment value was found to be 21.38, 21.52, and 15.25 respectively.

### Statistical analysis

All data were log2 transformed prior to statistical analysis. Sex was correlated with the expression of most target genes, so was included as a covariate where applicable. Linear regression including sex and disease status as covariates was used to test the association between gene expression and cell-type. Since the fibroblast samples were isolated only from males, the interpretation of this model can only be generalized to the male population. Linear regression was also used to test the association of disease in AECs adjusting for gender as a covariate. For fibroblasts, we did not adjust for any covariates and a *t*-test was employed to identify significantly different gene expression. All statistical analysis and figures were generated using the R software version 3.0.2 [37] and the ggplots2 package [38].

## Results

### Airway cell-specific expression of epigenetic modifying enzymes

**Fig. 1:**
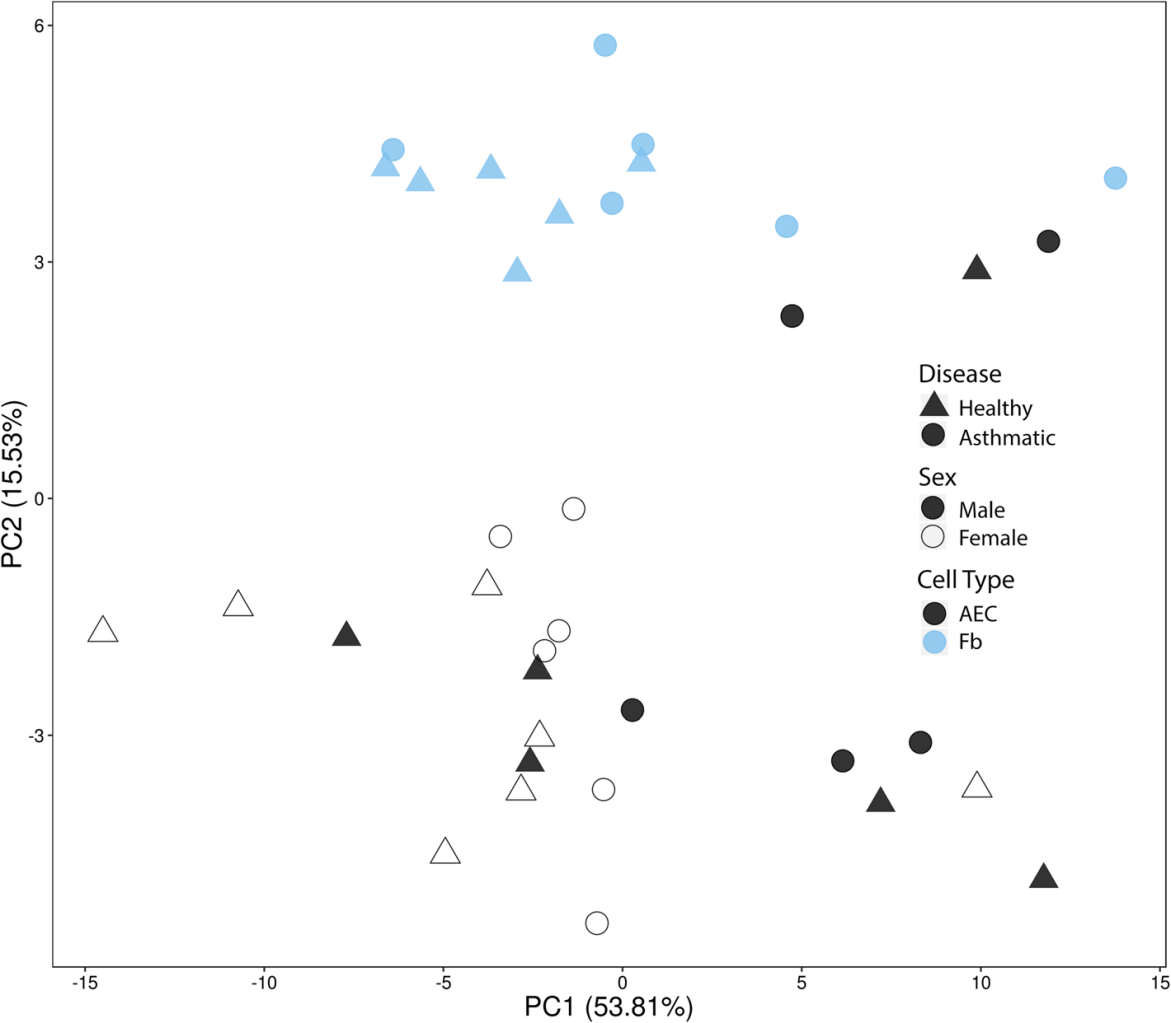
Principal component analysis (PCA) of epigenetic modifier enzymes in airway epithelial cells (AECs) and airway fibroblasts. Gene expression levels of 82 genes were used to construct the PCA plot. Healthy samples are identified with a triangle, asthmatic samples with a circle. Filled symbols indicate male samples whereas open symbols indicate female samples. AECs are shown in *black* and airway fibroblasts (Fb) are shown in *blue*

We profiled the expression of 82 genes across 5 families of epigenetic modifying enzymes in AECs and airway fibroblasts from healthy and asthmatic donors (Fig. 1). We observed that 53.81% of the variation across both cell types could be accounted for by the first principal component, PC2 accounted for 15.53% of the variation, and PC1 and PC2 combined explained 69.34% of the total variation in the data. Due to limitations in access to clinical characteristics of our cohort, we could not identify the variable resulting in the greatest source of variation PC1. However, given expression differences existed between cell types, we next proceeded to determine cell-specific gene expression profiles.

**Fig. 2:**
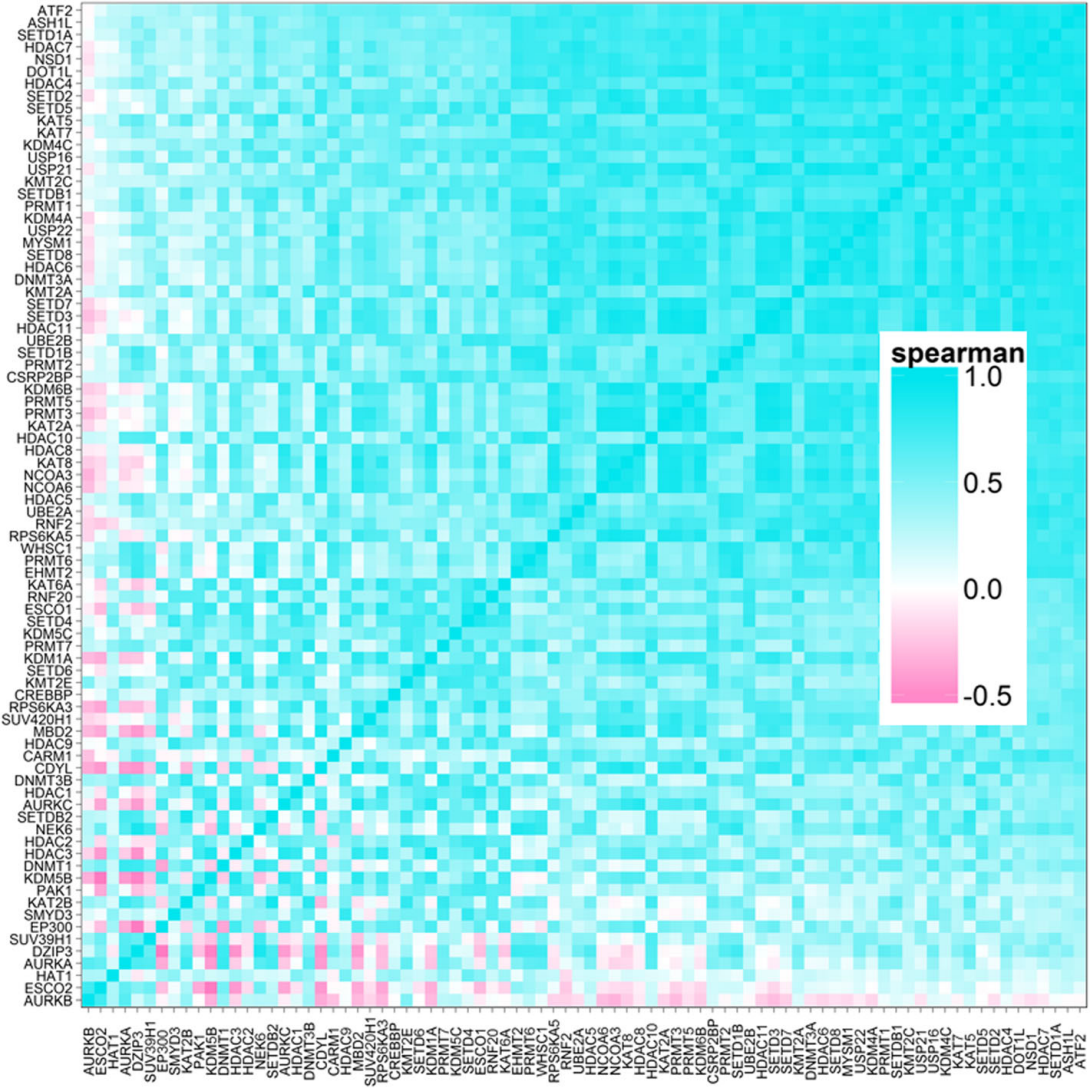
Co-expression heatmap of epigenetic modifying genes. Gene expression from both AECs and fibroblast cells from healthy individuals was used to analyze degree of co-expression of 82 genes involved in epigenetic mechanisms. Genes are listed on the x- and y-axis, *blue* indicates positive co-expression and *pink* indicates negative co-expression of genes

Co-expression analysis within healthy donors from AECs and fibroblasts showed that the epigenetic modifier enzyme genes which we examined were heavily co-expressed, with the majority showing positive co-expression (Fig. 2).

**Fig. 3:**
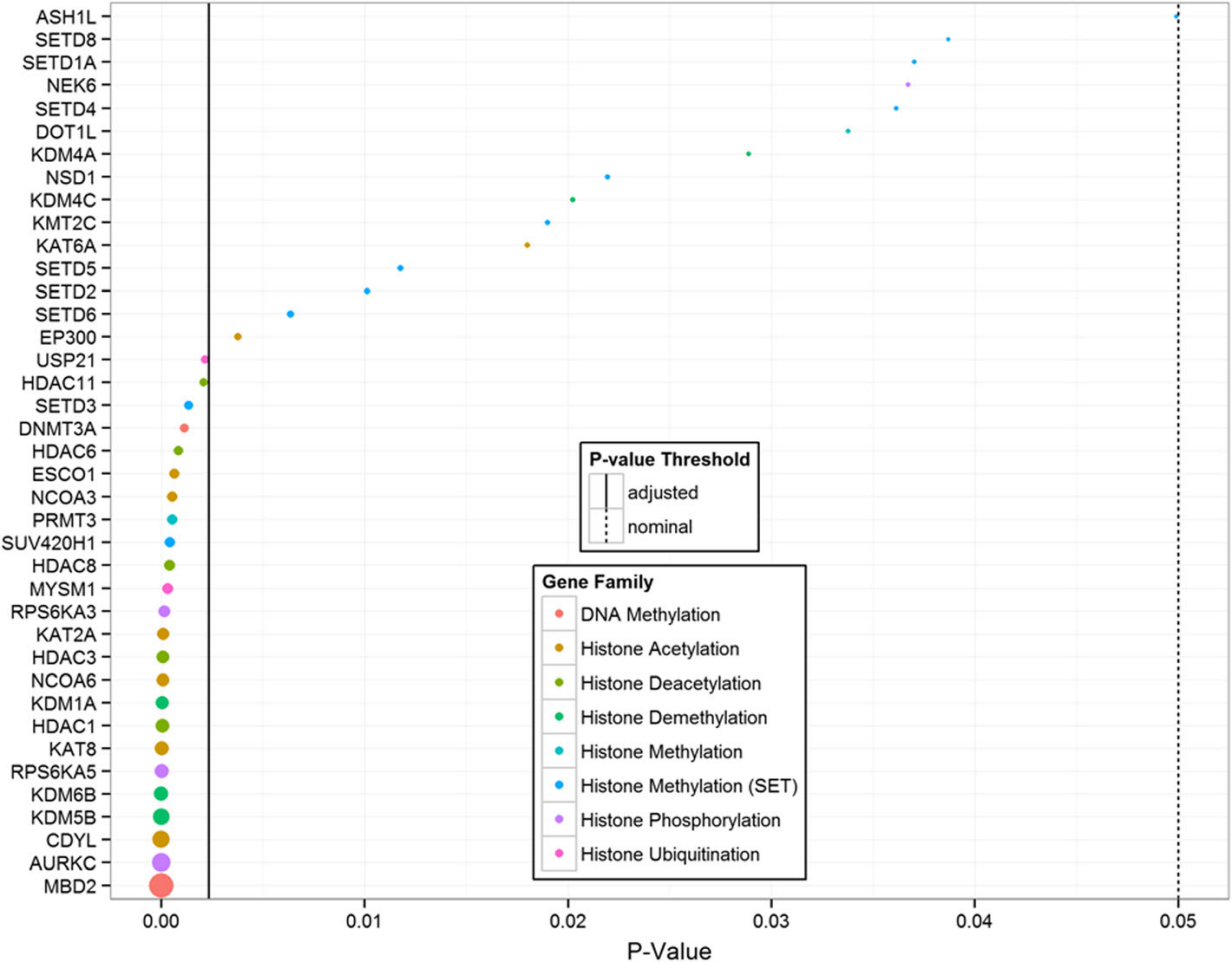
Differentially expressed epigenetic modifying genes in airway epithelial cells (AECs) compared to airway fibroblasts. Linear modeling was used to identify genes that were differentially expressed in AECs compared to airway fibroblasts. Genes are shown on the y-axis, *p*-values are shown on the x-axis. *Solid line* indicates significance threshold meeting ENIV criteria, *dotted line* indicates *p* = 0.05

Examination of differentially expressed genes between AECs and airway fibroblasts revealed 39 genes, of which 24 passed ENIV correction (Fig. 3 and Additional file 3: Table S3). Of the 24 genes, all showed increased expression in AECs as compared to airway fibroblasts. The differentially expressed genes were part of the DNA methylation (2 genes), histone methylation (6 genes), histone phosphorylation (3 genes), histone ubiquitination (2 genes), and histone acetylation (11 genes) families.

### Disease specific alterations in gene expression of epigenetic modification enzymes in airway epithelial cells

**Fig. 4:**
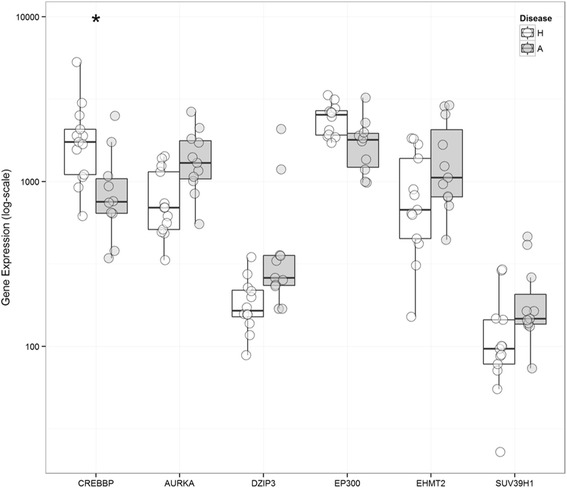
Differentially expressed epigenetic modifying genes in asthmatic compared to healthy airway epithelial cells (AECs). Healthy donors are shown in *white* whereas asthmatic donors are shown in *grey*. Linear regression was performed and found all 6 of these genes were significant, however only CREBBP met ENIV criteria. * indicates *p* < 0.05 after correction

To identify if asthma status influences epigenetic modifying enzymes, we compared the gene expression of the 82 genes in AECs derived from healthy and asthmatic donors (Additional file 4: Table S4). Although only CREBBP passed ENIV correction, linear regression identified differential expression of 6 genes: down regulation of the acetyltransferases CREBBP and EP300 and up regulation of the kinase AURKA, the ligases DZIP3 and the methyltransferases EHMT2 and SUV39H1 (Fig. 4).

**Fig. 5:**
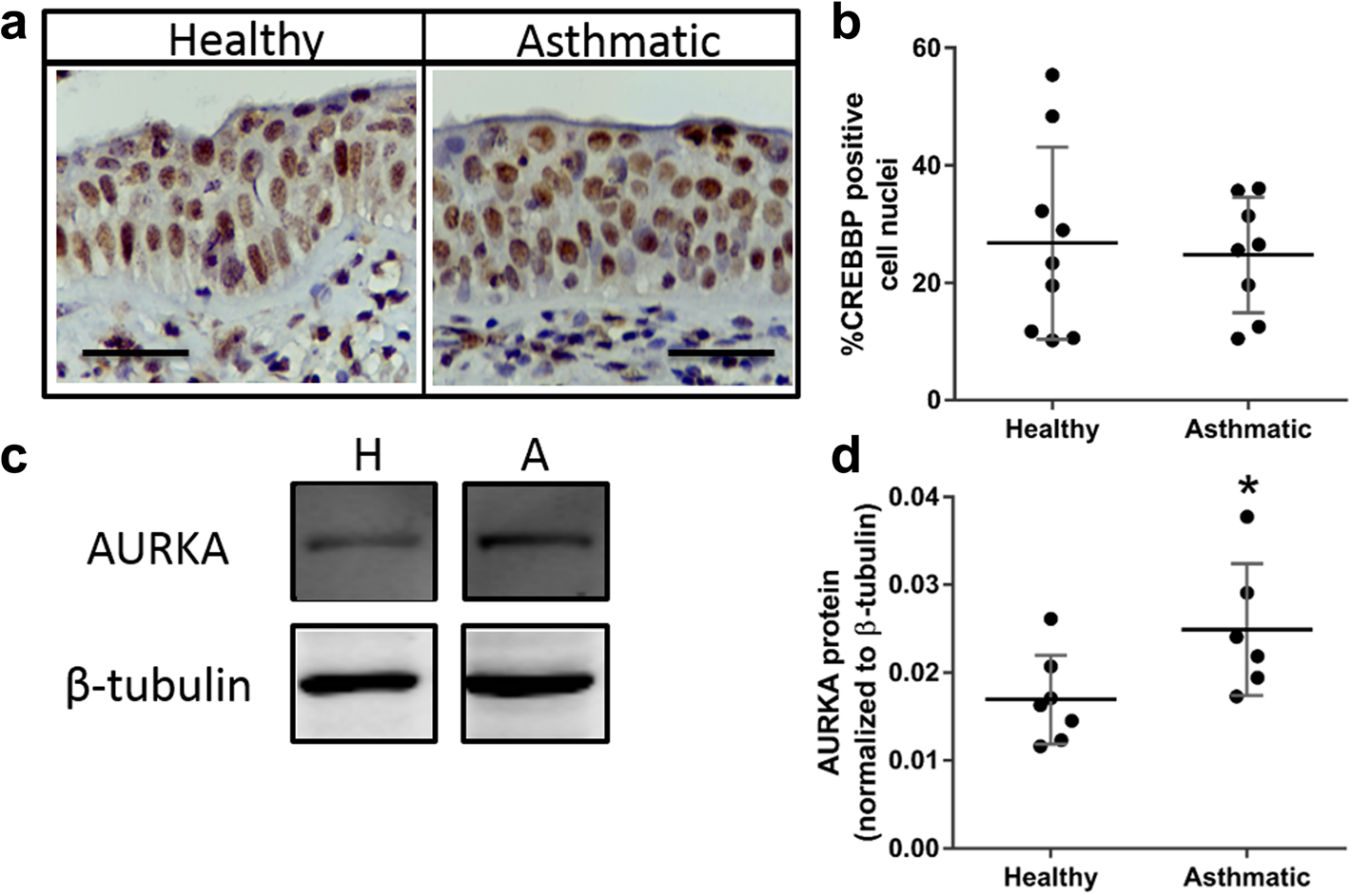
Expression of CREB-binding protein (CREBBP) and aurora kinase A (AURKA) in airway epithelial cells (AEC) from healthy and asthmatic donors. **a** CREBBP staining of formalin fixed, paraffin embedded airway sections from healthy and asthmatic donors. Scale bar is equal to 50 μm. **b** Data are presented as percent of CREBBP positive cell nuclei ± SD (*n* = 9 Healthy, *n* = 8 Asthmatic). **c** and **d** AURKA protein expression normalized to β-tubulin (± SD) in AECs from healthy (*n* = 7) and asthmatic donors (*n* = 6). A two tailed unpaired *t*-test was performed, * indicates *p* < 0.05

To confirm whether the observed changes in CREBBP and AURKA mRNA expression in AECs correspond to protein expression we performed immunohistochemistry and immunoblot. Although protein expression of CREBBP was not different between asthmatic (24.76 ± 3.47) and healthy donors (26.76 ± 5.46, *p* = 0.77, Fig. 5a and b), AURKA was significantly elevated in AECs from asthmatic (0.025 ± 0.003) as compared to healthy donors (0.017 ± 0.002, *p* = 0.04, Fig. 5c and d).

### Disease specific alterations in expression of epigenetic modification enzymes in airway fibroblasts

**Fig. 6:**
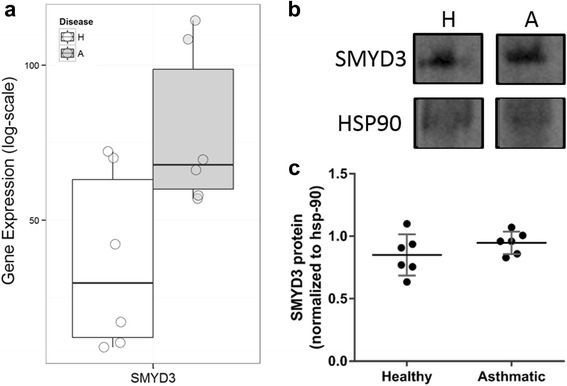
SET and MYND domain containing 3 (SMYD3) expression in asthmatic compared to healthy airway fibroblasts. SMYD3 expression was analyzed at the RNA (**a**) and protein (**b**) level in airway fibroblasts. **c** SMYD3 expression is normalized to HSP90 (± SD). For gene expression data, a *t*-test found SMYD3 to be significant however it did not pass ENIV correction. A two tailed unpaired *t*-test was performed on protein data (*n* = 6)

Next, we investigated if any differences existed in the 82 genes in airway fibroblasts isolated from healthy and asthmatic donors. We found increased mRNA expression of the histone methyltransferase SMYD3 in airway fibroblasts from asthmatics. However, this statistical significance did not pass ENIV correction (*p* = 0.02 and 0.37 after correction, Fig. 6a). When assessing protein expression of SMYD3, we found no significant difference in SMYD3 protein expression in airway fibroblasts isolated from healthy (0.85 ± 0.07) and asthmatic donors (0.94 ± 0.04, *p* = 0.23, Fig. 6b and c).

## Discussion

This is the first study to evaluate the gene expression levels of histone and DNA modifier enzymes in AECs and airway fibroblasts derived from human lung tissue. We found significantly higher expression for 24 of these enzymes in AECs compared to airway fibroblasts from healthy individuals. Further, we demonstrate that AURKA is differentially regulated in AECs from asthmatic compared to healthy donors. In addition, we identified a corresponding increase in AURKA protein expression in AECs from asthmatic compared to healthy donors, further supporting our findings. Even though AECs and fibroblasts reside in close proximity within the airway mucosa, the function of each cell is very different. These data support the notion that epigenetic modulation of gene expression may be important for cell type specificity, and may potentially influence susceptibility to diseases such as asthma.

Multiple studies have documented differential DNA methylation in relation to tissue and cell specificity, and how this is altered in diseases [31, 39–41]. Yet very few studies have focused on the global expression of enzymes responsible for DNA methylation expression. In our study, DNMT3a and MBD2 were both elevated in AECs compared to airway fibroblasts. DNMT3a is not only integral for mammalian development but also responsible for *de novo* DNA methylation [42]. It is possible that the elevated DNMT3a seen in AECs may reflect the cell’s geographical position. The airway epithelium is constantly in contact with external environmental factors thus must be responsive and adaptable to incoming stimuli. Elevated DNMT3a allows the cell to methylate genes *de novo* in response to these environmental stimuli. The increased expression of MBD2 may be a response to the increase in DNMT3a as MBD2 is a transcriptional repressor which binds methylated DNA [43]. To further support this theory, the complex which MBD2 forms to repress gene expression is not strongly bound to the DNA [43] suggesting a transient visit as would be expected from a responsive reaction.

The outcome of an epigenetic change can be variable depending on the particular modification that occurs. Methylation of lysine and arginine residues on histone tails is facilitated by enzymes which are specific to both residue and site yet the outcome can activate or repress transcription [13]. In contrast, histone acetylation, commonly associated with gene expression, is regulated by enzymes that have been described as promiscuous in their substrate specificity [14]. We identified differential expression of enzymes involved in both histone methylation and acetylation in AECs compared to airway fibroblasts. Of the 6 enzymes involved in histone methylation, half target the activating mark H3K4me; SETD3 methylates while KDM5B and KDM1A demethylate H3K4. This may indicate that AECs preferentially utilize H3K4 methylation over others to control gene expression. A similar observation was seen with histone acetylation as 5 HATs and 6 HDACs were identified. Three of the HDACs that were elevated in AECs comprise 75% of the class I HDAC family of enzymes important in controlling proliferation, differentiation, and tissue development programs [44]. Higher expression of the majority of the class I HDAC family of enzymes in epithelial cells may be a reflection of their considerable specialization as they have the capacity to differentiate and develop into a variety of epithelial cell types, which requires manipulation of the processes mentioned above.

We found elevated expression of 3 histone kinases and 2 DUBs when we compared AECs to airway fibroblasts. Although histone phosphorylation is commonly associated with gene activation, histone ubiquitination can result in both permissive and repressive states depending on the residue. However, all of the resulting histone modifications from the 5 above enzymes are associated with gene expression. This suggests there may be an imbalance in the regulation of these activating marks in AECs, potentially indicating lower levels of cellular transcriptional activity in airway fibroblasts compared to AECs.

Through its interaction with β-catenin, CREBBP has recently been identified as a pivotal component of the machinery maintaining an undifferentiated and proliferative state [45]. Inhibition of this interaction facilitates β-catenin and EP300 pairing which is thought to control cell differentiation [45, 46]. Our findings of decreased gene expression of CREBBP in AECs from asthmatics may indicate a divergence away from a proliferative state towards an initiated, but incomplete differentiation pathway. This imbalance of proliferation/differentiation mechanisms may contribute to the phenotypically immature epithelium seen in asthmatic airways.

In the context of disease, aurora kinases have been linked to spermatogenic arrest, chromosomal instability, and tumorigenicity in pathologies such as infertility, chronic inflammation, and a wide range of cancers [47–49]. AURKA is capable of phosphorylating H3S10, a site implicated in both gene activation and cell division [15, 50]. In a murine model of wound repair, rapid and sustained phosphorylation of H3S10 was associated with wound healing in intestinal epithelial cells [51]. Further, although the mechanism is not fully clear, phosphorylation of H3S10 is a critical component of chromatin compaction during mitosis [52]. Given that AECs from asthmatics are mitotically dyssynchronous [53], show defects in cell cycle regulation [54], and exhibit abnormal proliferation and delayed wound repair [55–57], our finding of increased AURKA expression may indicate aberrant regulation of these processes in asthma.

We identified elevated mRNA expression of the histone methyltransferase SMYD3 in airway fibroblasts from asthmatics. SMYD3 is integral to cell cycle regulation through interactions with RNA polymerase II and methylation of H3K4 [58]. In addition to gene activation through H3K4 methylation, SMYD3 is capable of gene repression through H2K20 methylation [59], suggesting a complex role for this enzyme. However, although differences in gene expression were seen, we were unable to replicate these findings at the protein level possibly indicating a further level of transcriptional control.

While we found many cell-specific and some disease specific changes in the enzymes involved in epigenetic modification, there are limitations to our study. We used a cell culture model that does not necessarily represent the complexity of cell – cell interactions known to be integral to airway mucosal homeostasis. However, a cell culture model allowed us to identify differences in the epigenetic modification families in relatively undifferentiated epithelial cells and fibroblasts under controlled conditions. Although we examined gene and protein expression of the epigenetic modifiers associated with asthma, we did not assess the activity of these enzymes, which has been shown to differ in disease. In addition, due to the sample size, we were unable to examine sex differences within our samples. Lastly, we did not look at the targeted epigenentic changes as a result of the differential expression of the epigenetic modifying enzymes and further studies would need to be performed to solidify the functional effects of the cell and disease specific changes we described in our cohort.

## Conclusions

In summary, we identified cell-specific variation in gene expression in each of the families of epigenetic modifying enzymes in AECs and airway fibroblasts. These data provide insight into the cell-specific variation in epigenetic regulation which may impact the functions of different cell types. We identified disease specific dysregulation of the histone kinase AURKA in AECs, which may play a role in processes important in the pathogenesis of asthma such as proliferation and inflammation. These findings provide further evidence of the importance of the epigenome in cell development and function.

## Additional files

Additional file 1: Table S1. Epigenetic modification genes including family, full name, and alias. (DOCX 19 kb)
Additional file 2: Supplementary Methods. (DOCX 24 kb)
Additional file 3: Table S3. Comparison of epigenetic modifier gene expression between epithelial cells and fibroblasts from healthy donors. (DOCX 17 kb)
Additional file 4: Table S4. Comparison of epigenetic modifier gene expression between airway epithelial cells from asthmatic and healthy donors. (DOCX 15 kb)

## Abbreviations

AEC: Airway epithelial cell
AURKA: Aurora kinase A
CREBBP: CREB binding protein
DNMT: DNA methyltransferase
DUB: Deubiquitinating enzyme
DZIP3: DAZ interacting zinc finger protein 3
EHMT2: Euchromatic histone-lysine N-methyltransferase 2
ENIV: Effective number of independent variables
EP300: E1A binding protein p300
Fb: Airway fibroblast
GAPDH: Glyceraldehyde-3-Phosphate Dehydrogenase
H2K20: Histone 2 Lysine 20
H3K4me: Histone 3 lysine 4 monomethylation
H3S10: Histone 3 serine 10
HAT: Histone acetyltransferase
HDAC: Histone deacetylase
HDM: Histone demethylase
HMT: Histone methyltransferase
HPRT1: Hypoxanthine phosphoribosyltransferase 1
KDM: Lysine (K)-specific demethylase
MBD2: Methyl CpG binding domain protein 2
PCA: Principal component analysis
RPL13A: Ribosomal protein L13a
SMYD3: SET and MYND domain containing 3
SUV39H1: Suppressor of variegation 3–9 homolog 1
