# Supplementary material for: Epigenetic modifying enzyme expression in asthmatic airway epithelial cells and fibroblasts
Source: BMC Pulm Med. 2017 Jan 31;17:24. doi: 10.1186/s12890-017-0371-0 (PMC5282738; doi:10.1186/s12890-017-0371-0)
Supplement: Additional file 1: Table S1. — Epigenetic modification genes including family, full name, and alias. (DOCX 19 kb) [file 12890_2017_371_MOESM1_ESM.docx]

Additional File 1

**Table S1. Epigenetic modification genes including family, full name, and alias.**

| Family | Gene | Name | Alias |
| --- | --- | --- | --- |
| DNA Methylation | DNMT1 | DNA methyltransferase 1 | DNMT, MCMT, AIM, CXXC9 |
|  | DNMT3A | DNA methyltransferase 3A |  |
|  | DNMT3B | DNA methyltransferase 3B | ICF |
|  | MBD2 | methyl-CpG binding domain protein 2 | DMTase, NY-CO-41 |
| Histone Demethylation | KDM1A | lysine (K)-specific demethylase 1A | AOF2, LSD1 |
|  | KDM5B | lysine (K)-specific demethylase 5B | JARID1B |
|  | KDM5C | lysine (K)-specific demethylase 5C | JARID1C |
|  | KDM4A | lysine (K)-specific demethylase 4A | JMJD2A |
|  | KDM4C | lysine (K)-specific demethylase 4C | JMJD2C |
|  | KDM6B | lysine (K)-specific demethylase 6B | JMJD3 |
| Histone Methylation | CARM1 | coactivator-associated arginine methyltransferase 1 | PRMT4 |
|  | DOT1L | DOT1-like histone H3K79 methyltransferase | KMT4 |
|  | EHMT2 | euchromatic histone-lysine N-methyltransferase 2 | G9A, BAT8 |
|  | KMT2A | lysine (K)-specific methyltransferase 2A | MLL |
|  | PRMT1 | protein arginine methyltransferase 1 | HRMT1L2 |
|  | PRMT2 | protein arginine methyltransferase 2 | HRMT1L1 |
|  | PRMT3 | protein arginine methyltransferase 3 | HRMT1L3 |
|  | PRMT5 | protein arginine methyltransferase 5 | HRMT1L5, SKB1 |
|  | PRMT6 | protein arginine methyltransferase 6 | HRMT1L6 |
|  | PRMT7 | protein arginine methyltransferase 7 |  |
|  | PRMT8 | protein arginine methyltransferase 8 | HRMT1L3, HRMT1L4 |
|  | SETDB2 | SET domain, bifurcated 2 | CLLD8, KMT1F, CLLL8, C13orf4 |
|  | SMYD3 | SET and MYND domain containing 3 | ZNFN3A1, ZMYND1, KMT3E |
| Histone Methylation (SET) | ASH1L | Ash1 (Absent, Small, Or Homeotic)-Like | KMT2H, ASH1 |
|  | KMT2C | lysine (K)-specific methyltransferase 2C | MLL3 |
|  | KMT2E | lysine (K)-specific methyltransferase 2E | MLL5 |
|  | NSD1 | nuclear receptor binding SET domain protein 1 | KMT3B, STO, ARA267 |
|  | SETD1A | SET domain containing 1A | KMT2F, SET1, SET1A |
|  | SETD1B | SET domain containing 1B | KMT2G, SET1B |
| Histone Methylation (SET) | SETD2 | SET domain containing 2 | KMT3A, HYPB, SET2 |
|  | SETD3 | SET domain containing 3 | C14orf154 |
|  | SETD4 | SET domain containing 4 | C21orf27, C21orf18 |
|  | SETD5 | SET domain containing 5 |  |
|  | SETD6 | SET domain containing 6 |  |
|  | SETD7 | SET domain containing 7 | KMT7, SET7 |
|  | SETD8 | SET domain containing 8 | KMT5A, SET8 |
|  | SETDB1 | SET domain, bifurcated 1 | KMT1E, ESET |
|  | SUV39H1 | suppressor of variegation 3-9 homolog 1 | KMT1A |
|  | SUV420H1 | suppressor of variegation 4-20 homolog 1 | KMT5B, CGI85 |
|  | WHSC1 | Wolf-Hirschhorn syndrome candidate 1 | NSD2, MMSET, TRX5 |
| Histone Phosphory-  lation | AURKA | aurora kinase A | STK15, STK6 |
|  | AURKB | aurora kinase B | STK12, AIM1, AIK2, ARK2 |
|  | AURKC | aurora kinase C | STK13, AIE2, AIK3 |
|  | NEK6 | NIMA-related kinase 6 |  |
|  | PAK1 | p21 protein (Cdc42/Rac)-activated kinase 1 | p65-PAK |
|  | RPS6KA3 | ribosomal protein S6 kinase, 90kDa, polypeptide 3 | MRX19, CLS, RSK2 |
|  | RPS6KA5 | ribosomal protein S6 kinase, 90kDa, polypeptide 5 | MSK1, RSKL |
| Histone Ubiquitination | DZIP3 | DAZ interacting zinc finger protein 3 | hRUL138 |
|  | MYSM1 | Myb-like, SWIRM and MPN domains 1 |  |
|  | RNF2 | ring finger protein 2 | BAP1, DING, HIPI3, RING1B |
|  | RNF20 | ring finger protein 20 | BRE1A |
|  | UBE2A | ubiquitin-conjugating enzyme E2A | RAD6A |
|  | UBE2B | ubiquitin-conjugating enzyme E2B | RAD6B, HR6B |
|  | USP16 | ubiquitin specific peptidase 16 |  |
|  | USP21 | ubiquitin specific peptidase 21 |  |
|  | USP22 | ubiquitin specific peptidase 22 | USP3L |
| Histone Acetylation | ATF2 | activating transcription factor 2 | CREB2, CREBP1, HB16 |
|  | CDYL | chromodomain protein, Y-like |  |
|  | CIITA | class II, major histocompatibility complex, transactivator | MHC2TA |
| Histone Acetylation | CSRP2BP | CSRP2 binding protein | KAT14, ATAC2, CRP2BP |
|  | ESCO1 | establishment of sister chromatid cohesion N-acetyltransferase 1 | EFO1, CTF |
|  | ESCO2 | establishment of sister chromatid cohesion N-acetyltransferase 2 | RBS, EFO2 |
|  | HAT1 | histone acetyltransferase 1 | KAT1 |
|  | KAT2A | K(lysine) acetyltransferase 2A | GCN5, GCN5L2 |
|  | KAT2B | K(lysine) acetyltransferase 2B | PCAF |
|  | KAT5 | K(lysine) acetyltransferase 5 | HTATIP, TIP60, ESA1 |
|  | KAT8 | K(lysine) acetyltransferase 8 | MYST1, MOF |
|  | KAT7 | K(lysine) acetyltransferase 7 | MYST2, ORC1, HBO1 |
|  | KAT6A | K(lysine) acetyltransferase 6A | MYST3, ZNF220, RUNXBP2 |
|  | KAT6B | K(lysine) acetyltransferase 6B | MYST4, MORF |
|  | NCOA1 | nuclear receptor coactivator 1 | KAT13A, SRC1, RIP160 |
|  | NCOA3 | nuclear receptor coactivator 3 | KAT13B, ACTR, AIB1, RAC3, SRC3, TRAM1 |
|  | NCOA6 | nuclear receptor coactivator 6 | PRIP, RAP250, ASC2, AIB3 |
|  | CREBBP | CREB binding protein | CBP, RSTS, KAT3A |
|  | EP300 | E1A binding protein p300 | p300, RSTS2, KAT3B |
| Histone Deacetylation | HDAC1 | histone deacetylase 1 | RPD3L1 |
|  | HDAC10 | histone deacetylase 10 |  |
|  | HDAC11 | histone deacetylase 11 |  |
|  | HDAC2 | histone deacetylase 2 | RPD3, YAF1 |
|  | HDAC3 | histone deacetylase 3 | RPD3-2, SMAP45 |
|  | HDAC4 | histone deacetylase 4 | AHO3, BDMR, HDACA |
|  | HDAC5 | histone deacetylase 5 | NY-CO-9 |
|  | HDAC6 | histone deacetylase 6 | CPBHM |
|  | HDAC7 | histone deacetylase 7 |  |
|  | HDAC8 | histone deacetylase 8 | CDLS5, RPD3, MRXS6 |
|  | HDAC9 | histone deacetylase 9 | MITR, HDAC7B, HDRP |
