# Supplementary material for: Epigenetic modifying enzyme expression in asthmatic airway epithelial cells and fibroblasts
Source: BMC Pulm Med. 2017 Jan 31;17:24. doi: 10.1186/s12890-017-0371-0 (PMC5282738; doi:10.1186/s12890-017-0371-0)
Supplement: Additional file 2: — Supplementary Methods. (DOCX 24 kb) [file 12890_2017_371_MOESM2_ESM.docx]

Additional File 2

Supplementary Methods

**Data Cleaning:** Cycle threshold (Ct) values were read on either ABI 7900 or ViiA7 PCR machines (Life Technologies). Values for which the Ct exceeded 35 or genes where >10% of readings were missing were omitted from analysis as these were considered unreliable. In total, 4 genes were removed consisting of CIITA, NCOA1, KAT6B, and PRMT8. The arithmetic mean was calculated for each gene from replicate samples. To facilitate downstream analysis, we used Grubb’s test to remove values considered to be outliers.

**Housekeeping Gene Selection:** Since we aimed to compare different sets of samples from different cell types, we needed to use separate normalization schemes. Thus, we selected a set of housekeeping genes separately for each cell-type. In each case, we employed a systematic method of selection which is based on a synthesis of various methods and recommendations (Table S2). We chose among a panel of five housekeepers: beta-actin (ACTB), glyceraldehyde-3-phosphate dehydrogenase (GAPDH), ribosomal protein L13a **(**RPL13A), hypoxanthine phosphoribosyltransferase 1 (HPRT1), and beta-2-microglobulin (B2M). Of the five available housekeeping genes, GAPDH and ACTB were removed from AEC normalization because they have previously been associated with asthma in epithelial cells [[1](#_ENREF_1)].

Table S2. **Housekeeping gene selection in airway epithelial and fibroblast cells.**

| Airway Epithelial Cells | | | | | | | | |
| --- | --- | --- | --- | --- | --- | --- | --- | --- |
|  | **Summary Statistics** | | **BestKeeper** | | **geNorm** | | **NormFinder** | |
| **Gene** | **SD** | **Rank** | **r** | **Rank** | **Mean M** | **Rank** | **Rho** | **Rank** |
| HPRT1 | 0.87 | 1 | 0.920 | 2 | 0.618 | 1 | 0.162 | 2 |
| RPL13A | 0.91 | 2 | 0.953 | 1 | 0.618 | 1 | 0.127 | 1 |
| B2M | 1.24 | 3 | NA | NA | 1.181 | 3 | 0.328 | 3 |
| Airway Fibroblast Cells | | | | | | | | |
| GAPDH | 0.36 | 1 | 0.888 | 2 | 0.498 | 1 | 0.104 | 1 |
| HPRT1 | 0.66 | 4 | 0.838 | 3 | 0.546 | 3 | 0.118 | 2 |
| ACTB | 0.73 | 5 | 0.940 | 1 | 0.498 | 1 | 0.161 | 4 |
| B2M | 0.62 | 3 | NA | NA | 0.768 | 5 | 0.152 | 3 |
| RPL13A | 0.50 | 2 | NA | NA | 0.718 | 4 | 0.204 | 5 |

Housekeeping gene stability was determined by summary statistics, BestKeeper, geNorm, and NormFinder algorithms. SD = standard deviation across subjects within the given cell-type. r = the correlation between the individual gene and the BestKeeper Index aggregated from the group of most correlated genes. NA = the correlation was too poor to use as part of the BestKeeper Index. Mean M = the stability value before removal at each iteration of the algorithm. Rho = the stability value obtained from the NormFinder algorithm.

First, we assessed the standard deviation and the normality of the distribution in the CT values of the candidate housekeepers, individually, with the idea that the best housekeeper should have a low-variance, symmetric distribution [[2](#_ENREF_2)]. In both cell types, all housekeepers but B2M were reasonably symmetric in distribution, though in general the sample size made it difficult to reject the assumption of normality and symmetry. In epithelial cells, HPRT1 was found to have the smallest variance, with RPL13A not much higher. In fibroblasts, GAPDH had by far the lowest variance. We also looked at whether the distribution of expression differed according to disease status or sex or correlated with age, and we did this both graphically and with simple statistical tests. Out of all comparisons, the only nominally significant result was sex and GAPDH in epithelial cells (p = 0.013).

Then, we combined several sets of correlated housekeepers into their geometric means according to the BestKeeper algorithm [[3](#_ENREF_3)] and called these BestKeeper indices (BKI). We looked at the variance of the BKIs for various combinations of housekeepers, and assessed housekeeper stability by looking at the correlation between individual housekeepers and the BKIs. We did this only in epithelial and fibroblasts samples separately. The best housekeeper or BKI was that which gave a smaller distribution and higher correlation with each BKI.

We also looked at geNorm [[4](#_ENREF_4)] in each cell type separately which calculates pair-wise stability of housekeeping genes using geometric mean. This algorithm gives a ranking of housekeepers according to Vandesompele’s M, a measure of pairwise stability, with the top genes appearing as a pair. Finally, we used NormFinder [[5](#_ENREF_5)] to identify housekeepers that were stable with respect to the variable of interest, disease. It is a model based approach that assigns a stability measure to each gene; high values of the stability measure correspond to low intergroup and intragroup variation. Both geNorm and NormFinder algorithms were applied using the R package NormqPCR v.1.12.0 available from Bioconductor [[6](#_ENREF_6)].

We selected a best set of housekeepers for each cell type (HPRT1 and RPL13A for epithelial cells, GAPDH for fibroblasts), and each was used to normalize the target gene expression for the asthma vs. healthy analyses within the cell types. For the cell-type comparison, we chose a housekeeping gene which performed reasonably well in both housekeeper selections (HPRT1).

References

1. He JQ, Sandford AJ, Wang IM, Stepaniants S, Knight DA, Kicic A et al. Selection of housekeeping genes for real-time PCR in atopic human bronchial epithelial cells. Eur Respir J. 2008;32(3):755-62. doi:10.1183/09031936.00129107.

2. Mane VP, Heuer MA, Hillyer P, Navarro MB, Rabin RL. Systematic method for determining an ideal housekeeping gene for real-time PCR analysis. J Biomol Tech. 2008;19(5):342-7.

3. Pfaffl MW, Tichopad A, Prgomet C, Neuvians TP. Determination of stable housekeeping genes, differentially regulated target genes and sample integrity: BestKeeper--Excel-based tool using pair-wise correlations. Biotechnol Lett. 2004;26(6):509-15.

4. Vandesompele J, De Preter K, Pattyn F, Poppe B, Van Roy N, De Paepe A et al. Accurate normalization of real-time quantitative RT-PCR data by geometric averaging of multiple internal control genes. Genome biology. 2002;3(7):RESEARCH0034.

5. Andersen CL, Jensen JL, Orntoft TF. Normalization of real-time quantitative reverse transcription-PCR data: a model-based variance estimation approach to identify genes suited for normalization, applied to bladder and colon cancer data sets. Cancer research. 2004;64(15):5245-50. doi:10.1158/0008-5472.CAN-04-0496.

6. Perkins JR, Dawes JM, McMahon SB, Bennett DL, Orengo C, Kohl M. ReadqPCR and NormqPCR: R packages for the reading, quality checking and normalisation of RT-qPCR quantification cycle (Cq) data. BMC genomics. 2012;13:296. doi:10.1186/1471-2164-13-296.
