# Supplementary material for: Epigenetic modifying enzyme expression in asthmatic airway epithelial cells and fibroblasts
Source: BMC Pulm Med. 2017 Jan 31;17:24. doi: 10.1186/s12890-017-0371-0 (PMC5282738; doi:10.1186/s12890-017-0371-0)
Supplement: Additional file 3: Table S3. — Comparison of epigenetic modifier gene expression between epithelial cells and fibroblasts from healthy donors. (DOCX 17 kb) [file 12890_2017_371_MOESM3_ESM.docx]

Additional File 3

Table S3. **Comparison of epigenetic modifier gene expression between epithelial cells and fibroblasts from healthy subjects.**

| Gene | AEC | Fb | p-value (uncor) | p-value (ENIV) | Family |
| --- | --- | --- | --- | --- | --- |
| MBD2 | 74276.62 | 30988.09 | 8.98E-09 | 1.92E-07 | DNA Methylation |
| AURKC | 467.73 | 127.18 | 6.40E-07 | 1.37E-05 | Histone Phosphorylation |
| CDYL | 18833.17 | 4158.65 | 1.57E-06 | 3.36E-05 | Histone Acetylation |
| KDM5B | 14083.06 | 5443.67 | 2.62E-06 | 5.60E-05 | Histone Demethylation |
| KDM6B | 8992.40 | 2328.83 | 1.27E-05 | 2.71E-04 | Histone Demethylation |
| RPS6KA5 | 2132.44 | 757.26 | 2.49E-05 | 5.33E-04 | Histone Phosphorylation |
| KAT8 | 8884.90 | 5036.50 | 2.76E-05 | 5.89E-04 | Histone Acetylation |
| HDAC1 | 24900.90 | 12862.58 | 4.60E-05 | 9.83E-04 | Histone Deacetylation |
| KDM1A | 18312.45 | 10693.99 | 5.21E-05 | 1.11E-03 | Histone Demethylation |
| NCOA6 | 8552.44 | 4511.20 | 6.98E-05 | 1.49E-03 | Histone Acetylation |
| HDAC3 | 16031.47 | 7794.09 | 7.05E-05 | 1.51E-03 | Histone Deacetylation |
| KAT2A | 11452.64 | 5328.93 | 1.07E-04 | 2.29E-03 | Histone Acetylation |
| RPS6KA3 | 7901.49 | 3961.21 | 1.55E-04 | 3.31E-03 | Histone Phosphorylation |
| MYSM1 | 7198.93 | 4410.64 | 3.09E-04 | 6.60E-03 | Histone Ubiquitination |
| HDAC8 | 5830.65 | 3535.27 | 4.05E-04 | 8.66E-03 | Histone Deacetylation |
| SUV420H1 | 15645.99 | 9959.45 | 4.22E-04 | 9.02E-03 | Histone Methylation (SET) |
| PRMT3 | 5676.43 | 3185.57 | 5.31E-04 | 1.14E-02 | Histone Methylation |
| NCOA3 | 10438.85 | 5939.99 | 5.36E-04 | 1.15E-02 | Histone Acetylation |
| ESCO1 | 5854.13 | 2982.52 | 6.24E-04 | 1.33E-02 | Histone Acetylation |
| HDAC6 | 5704.75 | 2970.15 | 8.48E-04 | 1.81E-02 | Histone Deacetylation |
| DNMT3A | 3821.05 | 1788.97 | 1.13E-03 | 2.42E-02 | DNA Methylation |
| SETD3 | 18067.48 | 11022.23 | 1.34E-03 | 2.87E-02 | Histone Methylation (SET) |
| HDAC11 | 2735.89 | 1635.21 | 2.09E-03 | 4.47E-02 | Histone Deacetylation |
| USP21 | 6155.87 | 3849.08 | 2.16E-03 | 4.62E-02 | Histone Ubiquitination |
| EP300 | 12148.16 | 8009.30 | 3.78E-03 | 8.09E-02 | Histone Acetylation |
| SETD6 | 1954.25 | 998.88 | 6.35E-03 | 1.36E-01 | Histone Methylation (SET) |
| SETD2 | 14833.84 | 10646.50 | 1.01E-02 | 2.16E-01 | Histone Methylation (SET) |
| SETD5 | 19863.11 | 13226.37 | 1.18E-02 | 2.51E-01 | Histone Methylation (SET) |
| KAT6A | 13340.33 | 10651.77 | 1.80E-02 | 3.85E-01 | Histone Acetylation |
| KMT2C | 3538.48 | 2591.61 | 1.90E-02 | 4.06E-01 | Histone Methylation (SET) |
| KDM4C | 1121.93 | 731.41 | 2.02E-02 | 4.32E-01 | Histone Demethylation |
| NSD1 | 9162.05 | 7582.80 | 2.19E-02 | 4.69E-01 | Histone Methylation (SET) |
| KDM4A | 9457.15 | 6983.03 | 2.89E-02 | 6.17E-01 | Histone Demethylation |
| DOT1L | 3095.34 | 1994.41 | 3.38E-02 | 7.22E-01 | Histone Methylation |
| SETD4 | 3294.10 | 2470.57 | 3.61E-02 | 7.73E-01 | Histone Methylation (SET) |
| NEK6 | 14479.50 | 27140.85 | 3.67E-02 | 7.85E-01 | Histone Phosphorylation |
| SETD1A | 6396.35 | 5516.00 | 3.70E-02 | 7.91E-01 | Histone Methylation (SET) |
| SETD8 | 12273.17 | 9604.35 | 3.87E-02 | 8.27E-01 | Histone Methylation (SET) |
| ASH1L | 6488.70 | 5540.87 | 4.99E-02 | 1.00E+00 | Histone Methylation (SET) |

The mean expression for epigenetic modifier genes is shown for fibroblasts (Fb) and epithelial cells (AEC). P-values are derived from linear modeling and are shown as uncorrected (p-value (uncor)) and corrected for the effective number of independent variables (p-value (ENIV)).
