# Supplementary material for: Epigenetic modifying enzyme expression in asthmatic airway epithelial cells and fibroblasts
Source: BMC Pulm Med. 2017 Jan 31;17:24. doi: 10.1186/s12890-017-0371-0 (PMC5282738; doi:10.1186/s12890-017-0371-0)
Supplement: Additional file 4: Table S4. — Comparison of epigenetic modifier gene expression between airway epithelial cells from asthmatic and healthy donors. (DOCX 15 kb) [file 12890_2017_371_MOESM4_ESM.docx]

Additional File 4

Table S4. **Comparison of epigenetic modifier gene expression between airway epithelial cells from asthmatic and healthy subjects.**

| Gene | A | H | p-value (uncor) | p-value (ENIV) | Family |
| --- | --- | --- | --- | --- | --- |
| CREBBP | 976.35 | 1950.32 | 1.32E-03 | 2.82E-02 | Histone Acetylation |
| AURKA | 1420.23 | 793.19 | 3.84E-03 | 8.22E-02 | Histone Phosphorylation |
| DZIP3 | 511.17 | 187.34 | 4.45E-03 | 9.50E-02 | Histone Ubiquitination |
| EP300 | 1746.94 | 2421.68 | 7.52E-03 | 1.61E-01 | Histone Acetylation |
| EHMT2 | 1455.07 | 899.63 | 2.32E-02 | 4.96E-01 | Histone Methylation |
| SUV39H1 | 202.99 | 121.04 | 3.13E-02 | 6.69E-01 | Histone Methylation (SET) |

The mean expression for epigenetic modifier genes is shown for AECs from healthy (H) and asthmatic (A) subjects. P-values derived from Student’s t-tests are shown as uncorrected (p-value (uncor)) and corrected for the effective number of independent variables (p-value (ENIV)).
